# Ether Versus Chloroform

**Published:** 1867-08

**Authors:** E. Seguin


### SELECTED ARTICLES.

---

### ARTICLE VIII.

### *Ether versus Chloroform.*

To the Editor of the Medical Record.

*Sir*—The death by chloroform which recently took place at Bellevue Hospital, gives a sad interest to the question of surgical anæthesia. The repeated accidents which have occurred in May, 1866, in Berlin; June, 1866, in Philadelphia; February, 1867 in New York, have naturally enough staggered the faith of many surgeons in the great anæsthetic.

Allow me, sir, to refresh the minds of your readers with reference to the past records of chloroform. As early as 1853, Baudens acknowledged eighty deaths, and A. Forzet found eighty-five. In 1859 Barrier de Lyon ascertained that there had been two hundred deaths. Diday collected from that date to 1864, twenty-one cases registered in England, leaving at least as many which were unrecorded. If there was another drug instrumental in the destruction of so many lives, would it not be ejected from the *materia*

*medica?* Truc, the fault has been put on the impurity of the article émployed; but how often has chloroform been used in case of accident, in its purity, as in the instance of Bellevue; showing that it need not borrow its toxic properties from heterogeneous substances. Hence, from 1847, the date of the beginning of the use of anæsthesia, surgeons have been divided into two classes, the chloroformists and etherists; and though the first-named had, at first, the advantage, their rivals have steadily persevered, patient and unrelenting, in their efforts to demonstrate the general efficiency and the absolute safety of ether.

In 1848, Cantu remarked that half of his chloroformized frogs died, and hardly any of his etherized ones. Sedillor admits, at the same date, that when he stops giving ether, anæsthesia may continue, but in no case become aggravated. Not so with chloroform; when discontinued after insensibility is produced, its action is continued, its symptoms may in some instances cause death. This circumstance constitutes the most marked difference between the anæsthetic rivals.

The few men who supported this view against triumphant chloroformists found an early and eminent representative in T. E. Petrequin, chief-surgeon of the Hotel de Lyon. For nearly twenty years he has banished chloroform and used ether in that hospital, the largest in France, where from fourteen to fifteen thousand patients are treated annually, and where more operations are performed than in any other. From this telling experience, Petrequin Diday, and in fact l'Ecole de Lyon, asserts that pure ether has accomplished in their hands, without accident, those services which chloroform has rendered elsewhere at a cost of several hundred lives. Is not this question worthy of further study? Yours, etc.,

E. Seguin, M. D.

—*The Medical Record.*

